# The Influence of Synaptic Size on AMPA Receptor Activation: A Monte Carlo Model

**DOI:** 10.1371/journal.pone.0130924

**Published:** 2015-06-24

**Authors:** Jesus Montes, Jose M. Peña, Javier DeFelipe, Oscar Herreras, Angel Merchan-Perez

**Affiliations:** 1 Departamento de Arquitectura y Tecnología de Sistemas Informáticos, Facultad de Informatica, Universidad Politecnica de Madrid, Madrid, Spain; 2 Laboratorio Cajal de Circuitos Corticales, Centro de Tecnología Biomédica, Universidad Politécnica de Madrid, Madrid, Spain; 3 Instituto Cajal, Consejo Superior de Investigaciones Científicas, Madrid, Spain; The Research Center of Neurobiology-Neurophysiology of Marseille, FRANCE

## Abstract

Physiological and electron microscope studies have shown that synapses are functionally and morphologically heterogeneous and that variations in size of synaptic junctions are related to characteristics such as release probability and density of postsynaptic AMPA receptors. The present article focuses on how these morphological variations impact synaptic transmission. We based our study on Monte Carlo computational simulations of simplified model synapses whose morphological features have been extracted from hundreds of actual synaptic junctions reconstructed by three-dimensional electron microscopy. We have examined the effects that parameters such as synaptic size or density of AMPA receptors have on the number of receptors that open after release of a single synaptic vesicle. Our results indicate that the maximum number of receptors that will open after the release of a single synaptic vesicle may show a ten-fold variation in the whole population of synapses. When individual synapses are considered, there is also a stochastical variability that is maximal in small synapses with low numbers of receptors. The number of postsynaptic receptors and the size of the synaptic junction are the most influential parameters, while the packing density of receptors or the concentration of extrasynaptic transporters have little or no influence on the opening of AMPA receptors.

## Introduction

Chemical synapses are fundamental elements in signal transmission in the mammalian brain. When a nerve impulse arrives at the presynaptic element (usually an axon terminal), synaptic vesicles fuse with a specialized region of the presynaptic membrane—the active zone (AZ)—and release neurotransmitter into the synaptic cleft. The neurotransmitter molecules then diffuse and interact with specific receptors located at the opposing postsynaptic membrane. The interaction between transmitter and receptor eventually triggers ion permeability changes and/or metabolic effects at the postsynaptic element (a dendritic spine, a dendritic shaft, a cell body or an axon). Specific receptors and other molecules accumulate at the postsynaptic density (PSD), so named because it appears as an electron-dense thickening of the membrane under the electron microscope [1, 2].

The availability of new electron microscopic methods permits the visualization, identification and segmentation of large numbers of synapses in three-dimensional samples of nervous tissue (e.g., [3, 4]). Critical geometrical features of synaptic junctions, such as their spatial distribution and size can then be extracted and measured. The size of 3D-reconstructed synaptic junctions can be measured using the diameter of the smallest sphere circumscribing the synaptic junction (Feret’s diameter). Statistical analysis of a large population of 3D-reconstructed synaptic junctions from all cortical layers in the rat somatosensory cortex has been carried out recently, showing that synaptic sizes follow a log-normal distribution [5, 6]. A more sophisticated method has also been developed to extract and measure the synaptic apposition surface, which is equivalent to the surface area of the AZ and the PSD [7]. This kind of measurement is relevant since it is known that the surface area of the AZ is proportional to the probability of synaptic vesicle release [8–10], while the surface area of the PSD is proportional to the total number of postsynaptic receptors (for AMPA receptors see, for example, [11–16]). This prompts the question of whether physiological features (such as the number of receptors that will be activated after the release of transmitter) can be inferred from morphological features of the synapses such as the size of the synaptic junction. However, both kinds of parameters (physiological and morphological) are very difficult to obtain in the same experimental setup and in a number of samples that is large enough to draw statistically sound conclusions. Simulation approaches are thus useful to assess the influence of different parameters on the various synaptic events at the molecular and ultrastructural levels. In this respect, Monte Carlo simulators are especially appropriate since they are capable of tracking the stochastic behavior of diffusing neurotransmitter molecules in a 3D intercellular environment and their interactions with synaptic receptors [17–19].

In this study, we have used the Monte Carlo simulator MCell [17, 20]. We performed simulations based on simplified models of glutamatergic synapses where AMPA receptors are present. We focused on the effects of different synaptic parameters on the number of receptors that open after release of a single synaptic vesicle with a fixed amount of transmitter. We excluded the variability in synaptic behavior that can be attributed to multivesicular release, release failure or other presynaptic variables. In this way, we focused on events that take place after the release of neurotransmitter and thus isolated the effects that several variables have on the strength and variability of the postsynaptic response. These variables included the number of postsynaptic receptors, the concentration of transporter molecules on extrasynaptic membranes, and the geometry of the synaptic junction. Our aim was to identify the most relevant parameters influencing the activation of AMPA receptors and to quantify these influences to allow the morphological features of the synapse to be related to its behavior.

## Materials and Methods

**Fig 1:**
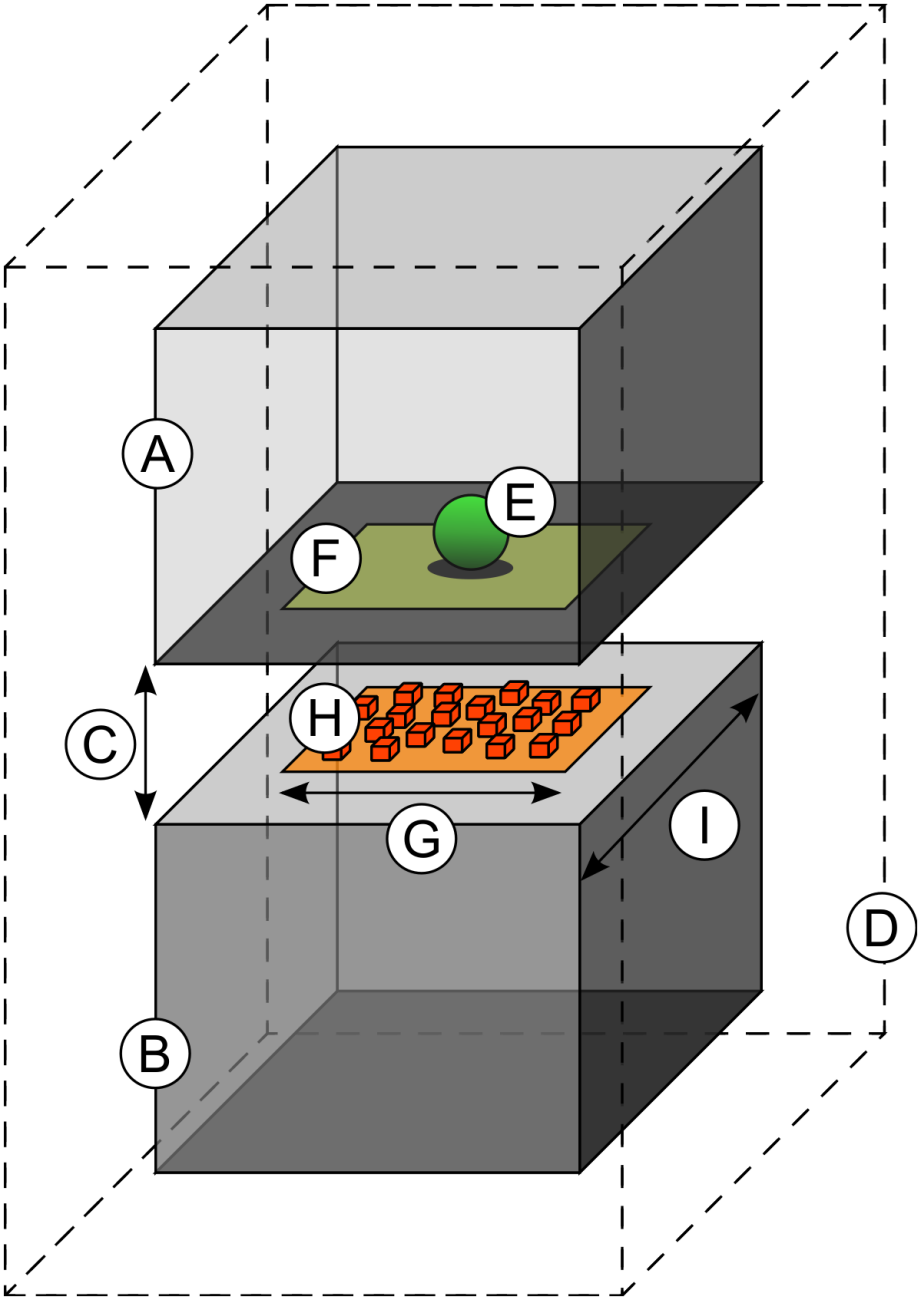
Geometrical model of chemical synapses. The presynaptic (a) and postsynaptic (b) elements are modeled as box-shaped structures separated by a distance representing the synaptic cleft (c). Perisynaptic membranes from neuronal and glial processes surrounding the synapse are represented by a larger box (d) that encloses both the pre- and postsynaptic elements. A single release site (e) is located at the center of the active zone (f). The PSD (g) is modeled as a square-shaped surface whose area is calculated from actual neocortical synaptic junctions. AMPA receptors (h) are located at the PSD at different concentrations. The total apposition of the pre- and post synaptic membranes is variable and extends beyond the synaptic junction (i).

We have performed simulations based on idealized models of excitatory synapses where AMPA receptors are present and the neurotransmitter involved is glutamate [21]. To explore the influence of synaptic size on the number of AMPA receptors that open after neurotransmitter release, we developed models with a simple geometry. In these idealized models (Fig 1), the pre- and post-synaptic elements (Fig 1a and 1b) were box-shaped structures that were separated by a gap of 20 *nm* (*H*_*c*_), representing the synaptic cleft (Fig 1c) [22]. For simplicity, we assumed that the shapes of neighboring cells were polyhedral [23] and we represented perisynaptic cell membranes as a box-shaped structure that enclosed the pre- and post synaptic elements (Fig 1d). A single vesicle release site (Fig 1e) was located at the center of the AZ (Fig 1f), which was represented in the presynaptic element with the same shape and size as the PSD [8, 24]. The PSD was represented in the postsynaptic element by a square of side length *L*_*s*_ (Fig 1g). We adjusted *L*_*s*_ to approximately represent the size of actual cortical synapses. To achieve this, we used a sample of 250 synaptic junctions that were three-dimensionally reconstructed from serial electron photomicrographs of layer III of the rat somatosensory cortex [7]. In this sample, we measured the Feret’s diameter (*D*_*F*_) as the diameter of the smallest sphere circumscribing the synaptic junction. We also measured the synaptic apposition surface (*SAS*), which is equivalent to the area of the AZ and the PSD (see details in [7]). We established the relationship between *L*_*s*_, *SAS* and *D*_*F*_ using the equation $L_{s}=\sqrt{SAS}=k\times D_{F}$. From the relationship between *D*_*F*_ and its corresponding *SAS* area in the 250 synaptic junctions, we calculated *k* = 0.624. Next, we modeled the probability distribution of *L*_*s*_ on the basis of the probability distribution of *D*_*F*_ in layer III described by Merchan-Perez et al., 2014 [5]. In that study, based on 1695 reconstructed synaptic junctions, the probability distribution of *D*_*F*_ was found to be a log-normal distribution with *μ* = 5.828 and *σ* = 0.446. To obtain a set of random values of *L*_*s*_ for our simulations, we sampled values using the above-mentioned probability distribution and translated them into *L*_*s*_ using the calculated *k* factor. The final values of *L*_*s*_ ranged from 60 to 825 *nm* and had a log-normal distribution with *μ* = 5.356 and *σ* = 0.446.

AMPA receptors were located in the PSD at different concentrations ranging from 500 to 3000 molecules per *μm* ^2^ [16, 25] (Fig 1h). We adopted the AMPA receptor kinetic model and rate constants described by Häusser and Roth, 1997 [26]. Glutamate transporter molecules (GluT) were located on the cell membranes of neuronal and glial elements surrounding the synaptic junction (Fig 1). The concentration of transporter molecules ([*GluT*]) ranged from 7000 to 12000 molecules/*μm* ^2^ [27]. We adopted the glutamate transporter kinetic model and rate constants described by Franks et al., 2002 [28]. The rate constants of AMPA and GluT kinetic models were adjusted to a temperature of 35°C using *Q*_10_ = 2.5 for both models [16, 29].

The apposition of cell membranes of the pre- and post-synaptic elements extended an additional distance in all directions outside the synaptic junction, exceeding the paired AZ and PSD. The side length of the total apposition of cell membranes (*L*_*a*_) (Fig 1i) was from 1 to 2 times the side length of the modeled synaptic junction. The distance between the perisynaptic box and the synaptic elements was set between 38 and 65 *nm* [30, 31], so that the volume representing the extracellular space was approximately 20% of the total volume [32, 33].

The main input variables in our experiments were *L*_*s*_, *L*_*a*_, [*AMPAr*] and [*GluT*]. Other relevant parameters such as the area of the PSD or synaptic area (*A*_*s*_) and the absolute number of AMPA receptors in the synapse (*nAMPA*) were derived from the main variables, as shown in Table 1.

**Table 1:** Modeling and simulation parameters.

**Parameter**	**Description**	**Values**
*H*_*c*_	Synaptic cleft height	20 *nm* [22]
*L*_*s*_	Side length of the PSD	60 to 825 *nm*, following a log-normal distribution with *μ* = 5.34 and *σ* = 0.45
*L*_*a*_	Side length of the total apposition of cell membranes	1 to 2 times the side length of the PSD [21]
*A*_*s*_ *	Synaptic area	*A*_*s*_ = *L*_*s*_^2^
[*AMPAr*]	AMPA receptor concentration	500 to 3000 receptors/*μm* ^2^ [16, 25]
[*GluT*]	Glutamate transporter concentration	7000 to 12000 molecules/*μm* ^2^ [27]
*nAMPA* *	Total number of AMPA receptors per synapse	*nAMPA* = [*AMPAr*] × *As* × 10^−6^
*N*_*g*_	Glutamate molecules per vesicle	3000 [31, 34, 35]
*D*_*g*_	Glutamate diffusion coefficient	0.33 *μm* ^2^/*ms* [36]
Δ*t*	Time step	1 *μs*
*T*	Total simulated time	10 *ms* (10,000 iterations)
*N*_*R*_	Number of simulation runs for each model	500

Asterisks (*) indicate parameters that were not actually used as input parameters for the simulations, but were calculated from them as shown.

Once the geometrical models had been built, the simulations were carried out with MCell software [20], exploiting the highly optimized Monte Carlo algorithms that it uses to track the stochastic behavior of diffusing molecules. Each simulation began with the release of the content of a synaptic vesicle at the center of the AZ. The vesicle was assumed to contain 3000 glutamate molecules [31, 34, 35]. We used a value of 0.33 *μm* ^2^/*ms* as an estimation of the diffusion coefficient of glutamate (*D*_*g*_) [36]. The receptor kinetic model assumes eight closed states and one open state [26]. Before the release of glutamate, all receptors were in the unliganded, closed state. After release we focused on the number of open receptors as a function of time since glutamate release. Modeling and simulation parameters are summarized in Table 1.

We randomly generated a total of 500 different synapses using different parameter configurations. For each of these configurations, *L*_*s*_ random values were obtained from a log-normal distribution, as explained above. Values for *L*_*a*_, [*AMPAr*] and [*GluT*] were obtained by independently sampling uniform distributions, each of them between the ranges indicated in Table 1. In this way, the sizes of our model synapses can be considered representative of the sizes of actual cortical synapses and other variables were set within plausible ranges (Table 1). We then simulated each of these synaptic configurations with MCell. Due to the stochastic nature of the simulations, each of the 500 configurations of synapses was simulated 500 times, thus generating a body of 250,000 raw simulation experiments. This gave a series of different results from a set of biologically based set ups with differences in aspects such as synaptic size, [*AMPAr*] and [*GluT*] [21]. Each simulation consisted of 10,000 iterations with a time step of 1 *μs*, corresponding to a total simulation time of 10 *ms* after neurotransmitter release (Table 1). The synaptic model simulations were performed using a supercomputer, the Magerit system, located at CeSViMA [37]. At the time of the experiments, Magerit was a computer cluster consisting of 245 eServer BladeCenter PS702 computer nodes, with a total of 3920 IBM PowerPC 3.3 *GHz* CPU cores and 7840 *GB* of RAM. The MCell developing team kindly provided a version of the MCell software for the PowerPC architecture. The simulation of synaptic models involved 250,000 jobs executed on this supercomputer, requiring approximately 1000 CPU hours. Since 500 CPUs were used simultaneously, the whole set of simulations took approximately 2 hours.

## Results

### Open AMPA receptor curves

**Fig 2:**
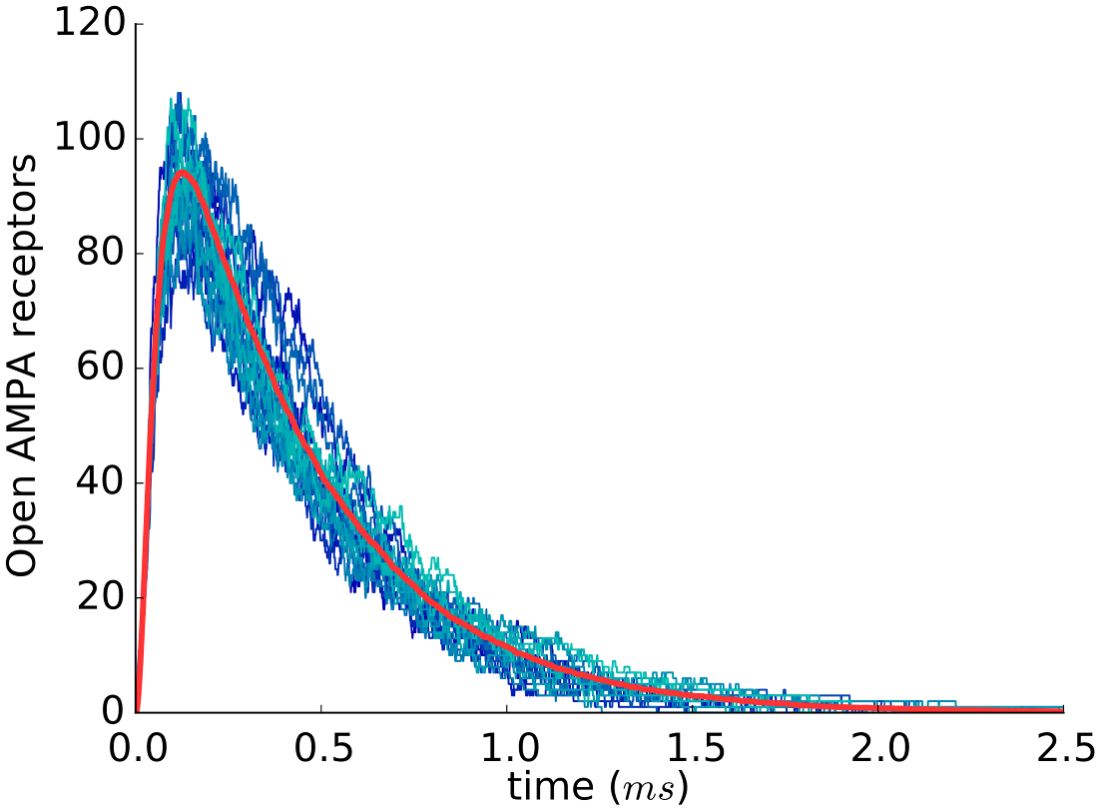
Number of open AMPA receptors after the release of a single vesicle of glutamate. An example of the curves obtained with Monte Carlo simulations of a single synapse configuration is shown. A total of 500 independent simulations of the same model synapse were run with different random seeds. For clarity, only 25 simulations have been represented (blue traces), showing the stochastic variability of the number of open AMPA receptors. The average curve is shown in red. In this example, synapse parameter values were: [*AMPAr*] = 2000 *molecules*/*μm* ^2^, [*GluT*] = 9500 *molecules*/*μm* ^2^, *L*_*s*_ = 450 *nm*, *L*_*a*_ = 675 *nm*.

When every synapse configuration had been simulated 500 times with different random seeds, the average number of open AMPA receptors was plotted as a function of time since glutamate release (Fig 2). Consistent with previous studies [28, 34, 38, 39], the curves obtained showed a rapid increment of the number of open receptors, followed by a progressively slower decrement, with a long tail descending towards 0.

For each of the 500 runs of every synapse configuration, we recorded the peak amplitude, representing the maximum number of AMPA receptors that are open simultaneously after glutamate release (*maxOPEN*), and the time taken to reach this peak. These two values (*maxOPEN* and peak time) give us a basic, yet powerful description of the curve. For these two variables, we calculated their average value (*μ*), standard deviation (*σ*) and coefficient of variation (*cv* = *σ*/*μ*) in the 500 runs. The latter (*cv*) provided us with a normalized measure of dispersion, independent of the variable scale. When all synapse configurations were considered, *maxOPEN* showed a mean value of 40.33 open AMPA receptors, with a *σ* and *cv* of 32.67 and 0.81, respectively. Mean *maxOPEN* values in the 500 model synapses ranged between 3 and 228 open AMPA receptors, suggesting the existence of high variability between individual synapses and indicating the critical relevance of some synapse parameters. In spite of this variability, the shape of the curves of open AMPA receptors in any given synapse was similar for different values of *maxOPEN* (Fig 2), and the total number of open AMPA receptors after glutamate release, measured as the area below the curve, showed a high correlation with *maxOPEN* (*r* = 0.998).

The peak time showed a mean value of 79.03 *μs*, with a *σ* and *cv* of 51.30 and 0.65, respectively, and average values in the 500 models ranged between 29 and 199 *μs*, indicating that all model synapses reached the peak amplitude within a very narrow band of less than 170 *μs*. We have not explored peak time further.

**Fig 3:**
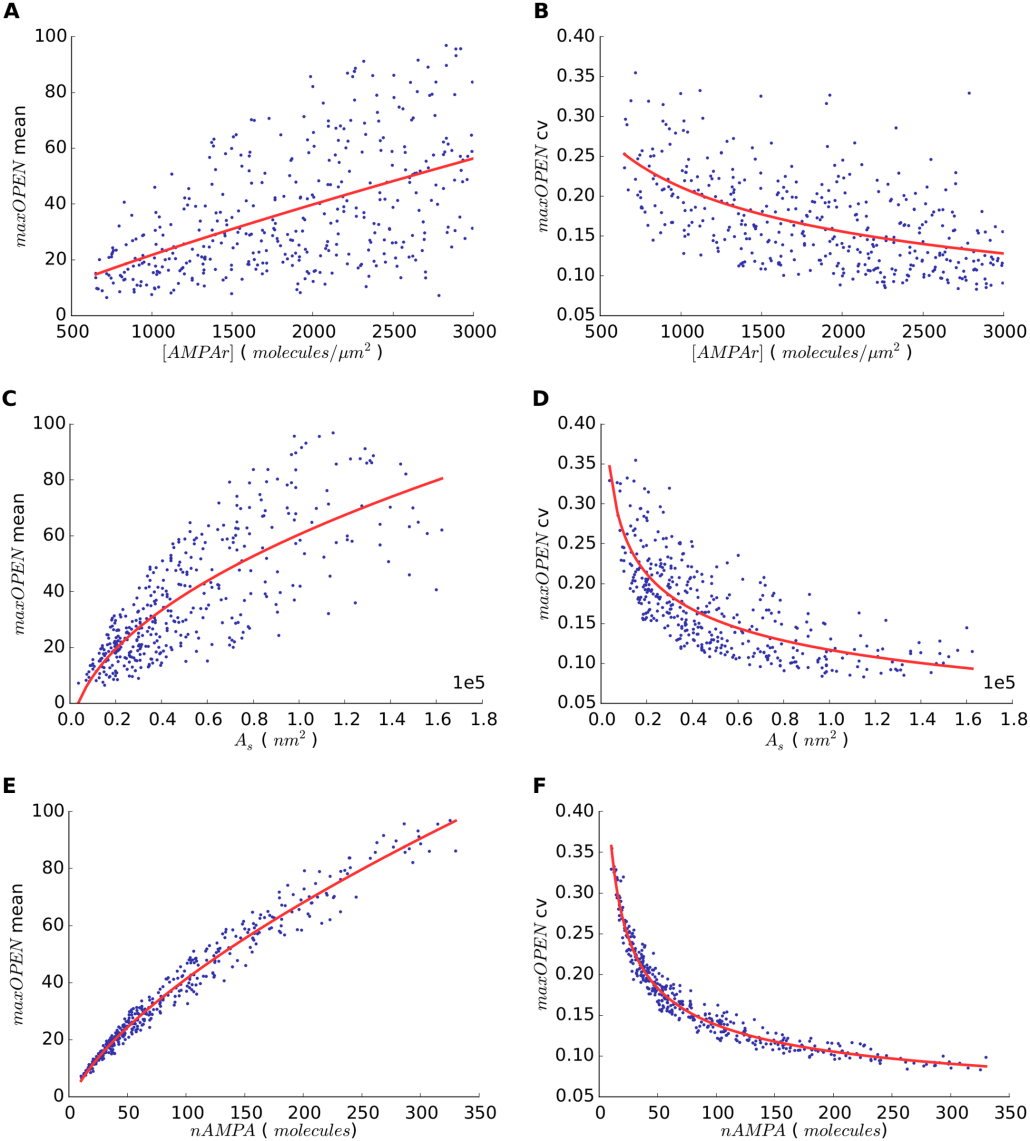
Relationship between synapse model parameters and open AMPA receptors. (a) Relationship between the AMPA concentration [*AMPAr*] in the model synapse and the maximum number of open AMPA receptors (*maxOPEN*) after the release of glutamate. Each point represents the mean of 500 simulations with different random seeds. (b) Relationship between [*AMPAr*] in model synapses and the coefficient of variation of *maxOPEN*. (c) and (d) show analogous values for synapse area *A*_*s*_. (e) and (f) use the total number of AMPA receptors *nAMPA* as base variable. The associated regression curves (red) have been represented for each dataset.

**Fig 4:**
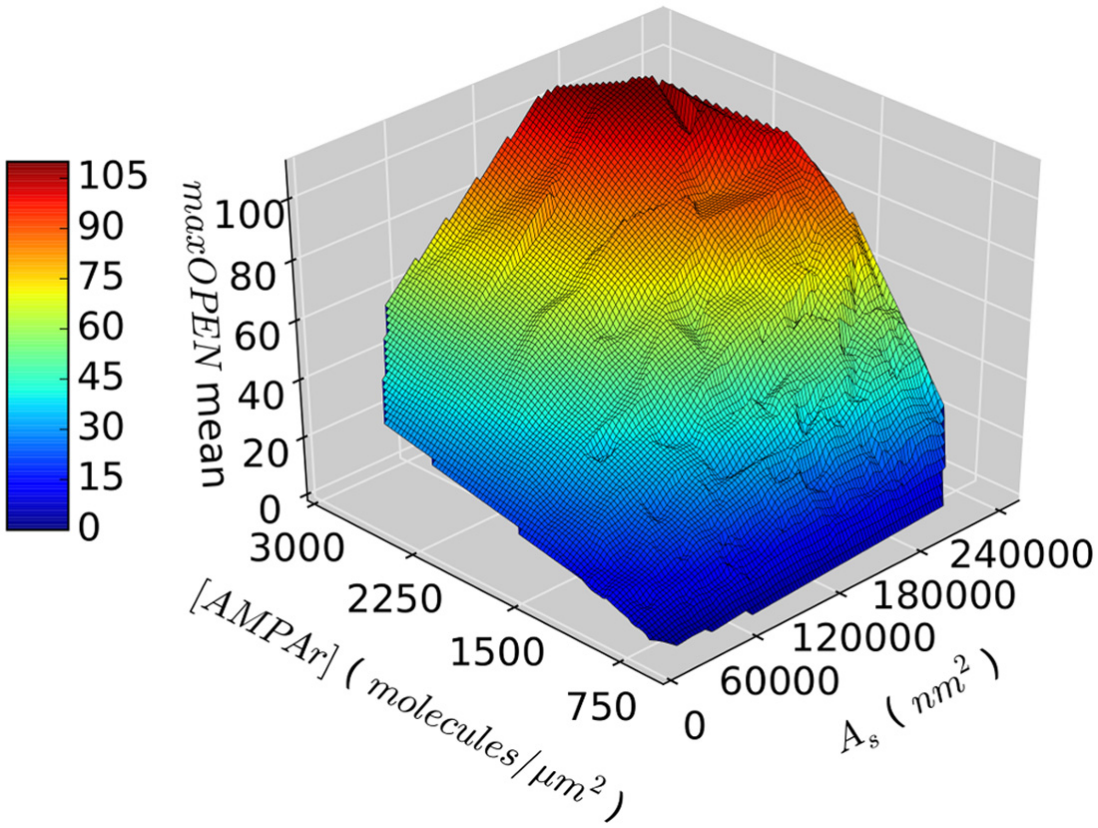
Relationship between the concentration of AMPA receptors in the PSD ([*AMPAr*]), the synaptic area (*As*) and the maximum number of open AMPA receptors (*maxOPEN*). Each data point represents the mean *maxOPEN* of 500 runs per synapse model, with different random seeds.

To assess the influence of the different simulation parameters on *maxOPEN*, we calculated the Pearson’s correlation coefficient (*r*) between them. The results are shown in Table 2. Some of the configuration parameters such as the concentration of glutamate transporters and the extension of the apposition of cell membranes outside the synapse showed no correlation with *maxOPEN* (∣*r*∣ < 0.04). Interestingly, the concentration of AMPA receptors [*AMPAr*] in the PSD showed only a weak correlation with *maxOPEN* and its *cv* (*r* ≈ 0.5). By contrast, synapse size (measured either as *L*_*s*_ or *A*_*s*_) showed a relatively strong correlation with *maxOPEN* (0.75 < *r* < 0.8) and the absolute number of receptors present in the synapse (*nAMPA*) showed the highest correlation (*r* = 0.98). Fig 3 shows scatter plots of mean and *cv* *maxOPEN* in model synapses with different [*AMPAr*], *A*_*s*_ or *nAMPA*. The plots show that *maxOPEN* tends to increase with all three parameters, although this tendency is much clearer with *A*_*s*_ and *nAMPA*, with no apparent saturation effects (Fig 3a, 3c and 3e). In our simulations, *nAMPA* depends on the combination of [*AMPAr*] and *A*_*s*_, so the highest peak amplitudes can only be obtained by having both a large synapse (large *A*_*s*_) and a high receptor concentration (high [*AMPAr*]), while none of these parameters alone suffices to evoke a strong response (Fig 4).

**Table 2:** Linear correlation between synapse configuration parameters and the mean and cv of the maximum number of open AMPA receptors (*maxOPEN*).

**Variable**	*maxOPEN* mean	*maxOPEN* *cv*
[*GluT*]	-0.033	0.017
*L*_*a*_	-0.036	0.047
[*AMPAr*]	0.530	-0.548
*A*_*s*_	**0.775**	-0.680
*L*_*s*_	**0.790**	-0.729
*nAMPA*	**0.980**	**-0.825**

Pearson’s correlation coefficients greater than 0.75 or lower than −0.75 have been highlighted in **bold**.

It is also interesting to note the inverse correlations of [*AMPAr*], synaptic size (*L*_*s*_ or *A*_*s*_) and *nAMPA* with the *cv* of *maxOPEN* (Table 2). The *cv* was highest with small [*AMPAr*], synaptic size or low *nAMPA* and then rapidly decreased and tended to stabilize (Fig 3b, 3d and 3f). This effect was especially clear with *A*_*s*_ and *nAMPA*, and seemed to indicate that the smaller the synapse or absolute number of receptors, the higher the variability of the synaptic response and *vice versa*.

**Fig 5:**
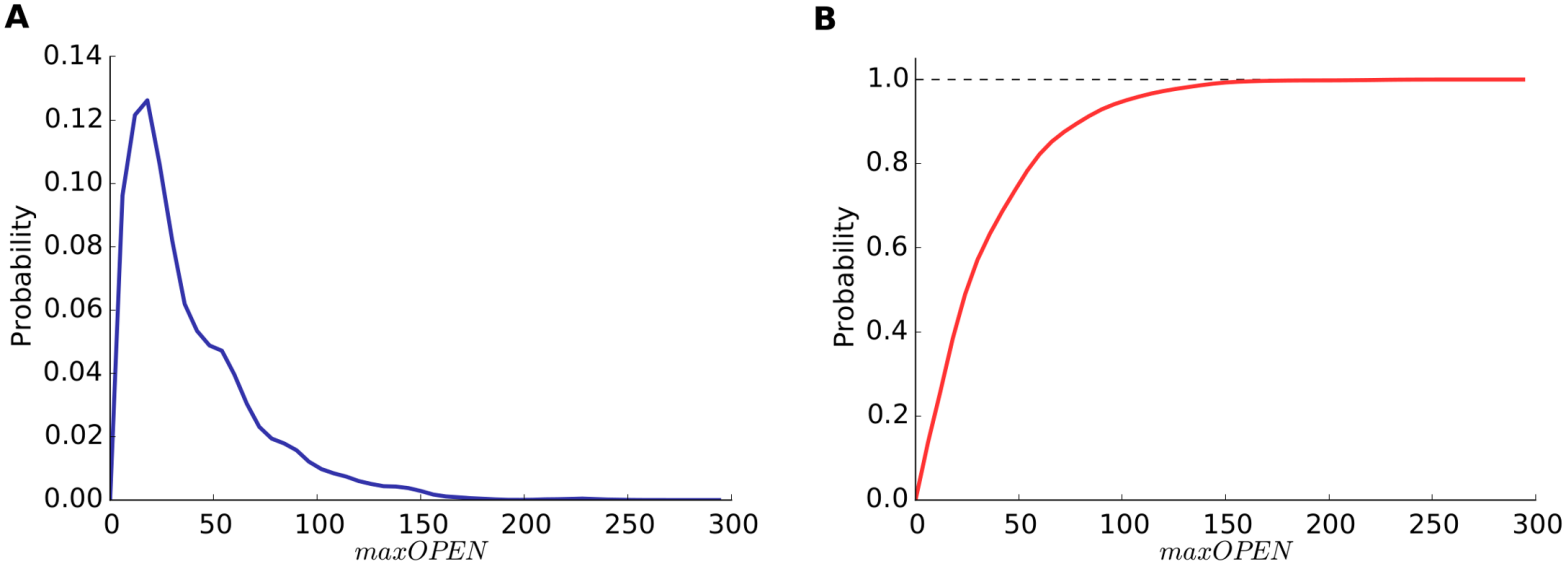
Probability functions of the maximum number of open AMPA receptors. The probability density function (a) and the cumulative distribution function (b) have been calculated from the 250,000 simulations executed as base experiments. The probability density function shows a rapid increase to a mode value of 21 open receptors and a slower decrease. The cumulative distribution shows a sharp increase such that in most cases (90%) *maxOPEN* was 100 or less open receptors.

**Fig 6:**
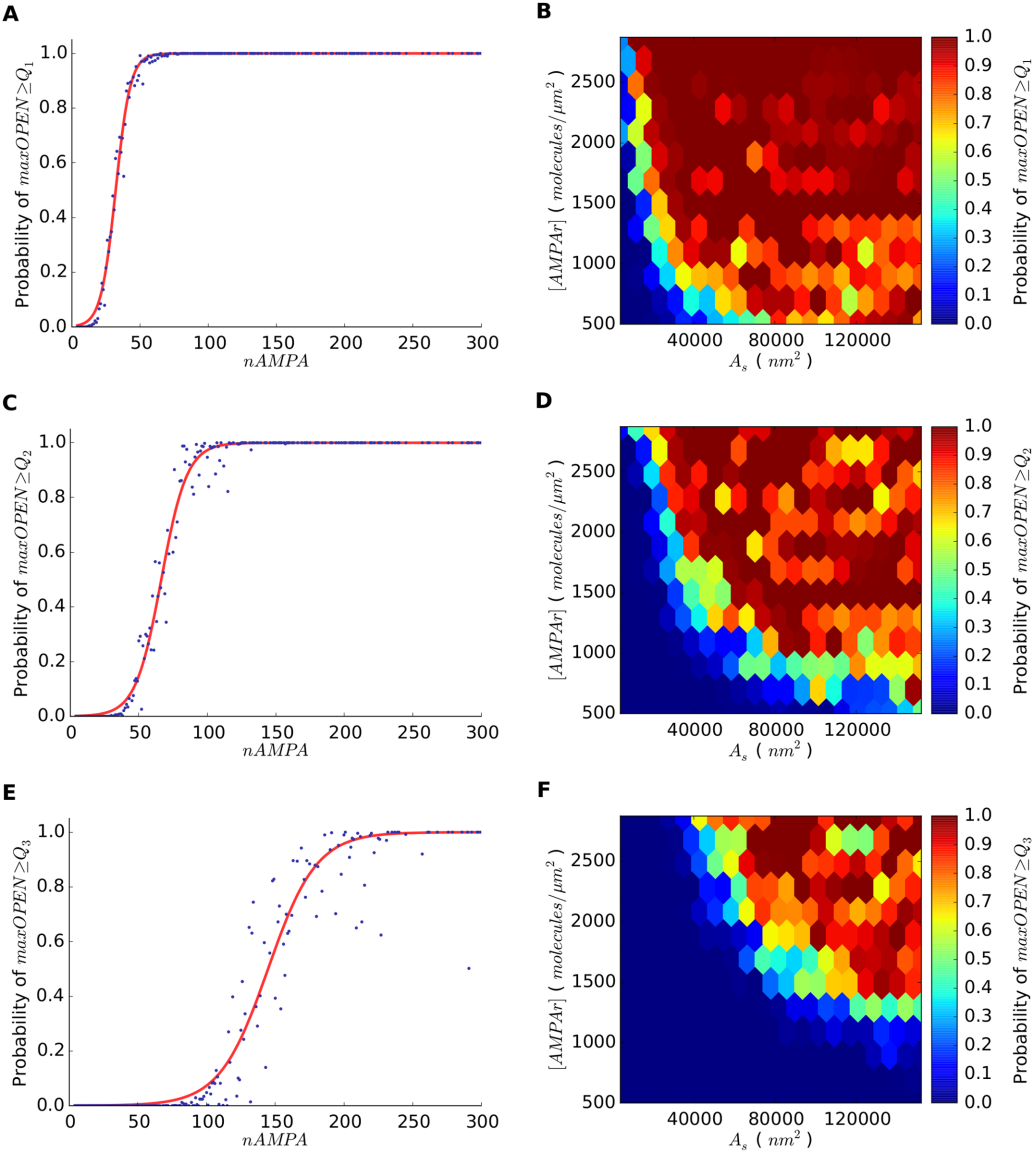
Synapse activation probabilities. Probability of having a maximum number of open AMPA receptors (*maxOPEN*) greater or equal to the quartile values *Q*_1_ = 17; *Q*_2_ = 30 and *Q*_3_ = 55. Since the total number of receptors in the synapse (*nAMPA*) is a combination of [*AMPAr*] and *A*_*s*_, probability values are shown with respect to *nAMPA* separately (a, c, e), and [*AMPAr*] and *A*_*s*_ combined (b, d, f).

To further explore *maxOPEN*, we also calculated its probability density function and cumulative distribution in the 250,000 simulations (Fig 5). The probability density function showed a rapid increase to a mode value of 21 open receptors (frequency of 0.125) and a slow decrease. The cumulative distribution showed a sharp increase such that in most cases (90%) *maxOPEN* was 100 or less open receptors. We also calculated the three quartile values of the distribution of *maxOPEN* (*Q*_1_, *Q*_2_ and *Q*_3_), which were 17, 30 and 55 open receptors, respectively. Since the opening of a receptor is a stochastic phenomenon, any given synapse will have different *maxOPEN* in different runs of the simulation. Therefore, synapses with any set of fixed parameters will have a different probability of being inside each quartile interval. In practice, we calculated the probability of synapses having a value of *maxOPEN* higher than *Q*_1_, *Q*_2_ and *Q*_3_. This analysis is relevant if we assume that the strength of the postsynaptic response depends on the total number of open receptors (see discussion). These probabilities were calculated from our simulations with respect to different synapse configuration parameters such as [*AMPAr*], *A*_*s*_, and *nAMPA* (Fig 6).

### Prediction of the peak of open AMPA receptors from the size of the synapse or the number of receptors

Having determined that the synaptic surface area (*A*_*s*_) and especially the total number of AMPA receptors in the PSD (*nAMPA*) are correlated with the peak amplitude of open AMPA receptors (*maxOPEN*), we next tried to ascertain whether knowing the value of *A*_*s*_ or *nAMPA* in a given synapse would allow us to predict *maxOPEN*, and how accurate this prediction would be. The value of [*AMPAr*] alone was not included in this part of the study, since the results presented showed no strong correlation with *maxOPEN* (see Table 2 and Fig 3a and 3b). To estimate, as accurately as possible, the mathematical relationship between *A*_*s*_ and *nAMPA* and the peak open AMPA curve variables, we selected a standard power function with three coefficients (Eq 1) as the simplest regression function to fit our data. We calculated the value of the coefficients of this function for each curve variable using the *Nonlinear Least Squares* curve fitting technique (nls) [40, 41] provided by the R statistical software [42]. These values can be found in Table 3.

$$f\left(x\right)=ax^{b}+c$$ (1)

**Table 3:** Coefficients of the regression functions, for *A*_*s*_ and *nAMPA* vs. each curve variable.

**Curve**	**a**	**b**	**c**
*A*_*s*_ vs. *maxOPEN* mean	0.278	0.487	-14.90
*A*_*s*_ vs. *maxOPEN* *cv*	2.300	-0.184	-0.159
*nAMPA* vs. *maxOPEN* mean	2.175	0.663	-4.661
*nAMPA* vs. *maxOPEN* *cv*	0.990	-0.457	0.017

These coefficients correspond to a regression function of the form *f*(*x*) = *ax* ^*b*^ + *c*.

Once we obtained the regression curves, we wanted to determine how well they fitted the simulation data. To achieve this, we used Root Mean Squared Error (*RMSE*) and the coefficient of determination (*R* ^2^). This coefficient provides a measure of how well a regression model fits the data. *R* ^2^ usually has a value between 0 and 1 (sometimes it can be less than 0), where 1 indicates an exact fit to the reference data and a value less than or equal to 0 indicates no fit at all.

We calculated both *RMSE* and *R* ^2^ for each of the regression curves previously described. Nevertheless, these values measure how well our regression model fits the original data, that is, the same data we used to calculate it. As a consequence, our model may be valid only for this specific data set, and therefore would not represent the general pattern, but rather only the specific scenarios contained in it. This is an undesired phenomenon usually called *overfitting*. To ensure that our regression model was not *overfitted* to the data that was used to produce it, we needed to compare it to a new data set, typically called a test set. For our test set, we generated a new independent dataset from a series of 100 new synapse simulation configurations, using the same procedure described in Materials and Methods. These new simulations were never used during the regression model construction, and were produced for validation purposes only. Using this test data set, we calculated the accuracy of our regression models, using the same metrics as before. As shown in Table 4, *RMSE* and *R* ^2^ values were similar for the original training dataset and the newly generated dataset, so this final validation rules out the possibility of *overfitting*. The results showed that, of the synapse parameters examined, the one that best predicts *maxOPEN* mean and *cv* is the number of AMPA receptors (*nAMPA*). *A*_*s*_ alone has a moderate predictive value for *maxOPEN* mean and *cv*, while [*AMPAr*] alone has no predictive value, as explained before.

**Table 4:** Curve fitness, for *A*_*s*_ and *nAMPA* vs. *maxOPEN* mean and *cv* in the simulation training dataset and in a newly generated independent test dataset.

	**Training dataset**		**Test dataset**	
**Curve**	***RMSE***	***R*^2^**	***RMSE***	***R*^2^**
*A*_*s*_ vs. *maxOPEN* mean	13.209	0.624	13.553	0.489
*A*_*s*_ vs. *maxOPEN* *cv*	0.036	0.579	0.052	0.327
*nAMPA* vs. *maxOPEN* mean	3.485	0.974	3.425	0.967
*nAMPA* vs. *maxOPEN* *cv*	0.012	0.957	0.016	0.938

### Receptor activation as a function of distance to release site

**Fig 7:**
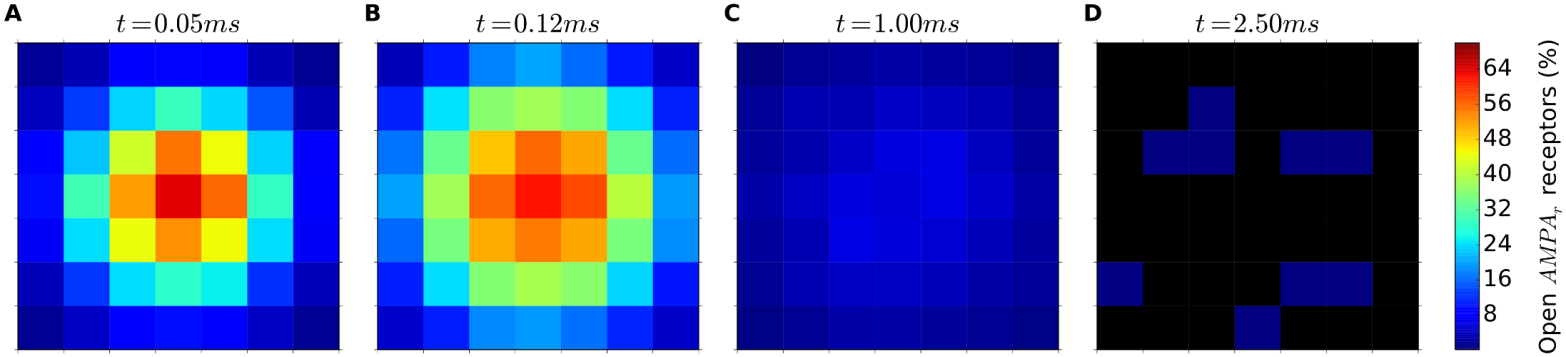
Percentage of open AMPA receptors at different positions of the PSD. Panels a to d represent the PSD of a model synapse at different time points after a single vesicle of glutamate is released at the center of the synapse. The PSD has a total side length of 385 *nm*; it has been divided into cells with a side length of 55 *nm* and the percentage of open AMPA receptors within each cell has been represented by a color scale. Time points correspond to an early stage after glutamate release (a), the peak of open AMPA receptors (b), a time point after the peak (c) and a late stage when open receptors are scarce or absent (d). Although open receptors are preferentially located near the release point, all receptors, including the most peripheral ones, contribute to the synaptic response.

Previous studies have shown that the opening probability of AMPA receptors decrease with distance to release site [38], suggesting that AMPA response results from a “hot spot” of open receptors located close to the release site, with negligible contribution of distant receptors [43]. If peripheral receptors do not contribute to synaptic response, the influence of synaptic size would be smaller than we have observed in our experiments, while the relevance of the density of AMPA receptors would be higher than observed. To rule out this possibility, we performed additional experiments to calculate the opening probability of AMPA receptors as a function of distance to the release site. We selected a particularly large synapse, in order to better detect if the most peripheral receptors reach the open state even in such an extreme scenario. The simulation parameters of this configuration were: *L*_*s*_ = 385 *nm*, *L*_*a*_ = 578 *nm*, [*AMPAr*] = 1750 *receptors*/*μm* ^2^, [*GluT*] = 9500 *transporters*/*μm* ^2^ (see Table 1 for details). Fig 7 shows the results of these simulations. The opening probability is highest close to the release site. However, Fig 7 also shows that all receptors in the PSD, including the most peripheral ones, have a non-zero probability of opening before, during and after the peak of receptor activation (Fig 7a, 7b and 7c, respectively). More specifically, at the instant of peak activation (Fig 7b), where maxOPEN is reached, even the most peripheral regions of the PSD show a receptor activation of approximately 10% or higher. This can be explained by the high mobility of glutamate (with a diffusion coefficient of 0.33 *μm* ^2^/*ms*), which reaches even the farthest receptors very quickly. Only at later stages does the opening probability decrease to zero in the whole PSD (Fig 7d).

## Discussion

For the simulations in the present study, we have used the actual sizes and shapes of a large number of cortical synaptic junctions that were 3D-reconstructed from electron microscope serial images [5, 7]. Our results obtained with the statistical analyses of previously generated Monte Carlo computational simulations of synapses suggest that the maximum number of receptors that will open after the release of a single synaptic vesicle may show a ten-fold variation in the whole population of synapses. Moreover, when individual synapses are considered, there is also a stochastical variability that is maximal in small synapses with a small number of AMPA receptors. This source of synaptic variability is independent of synaptic fluctuations due to variations of quantal size and release probability, since these factors were maintained constant in our simulations.

The number of AMPA receptors at the PSD is the most important parameter determining the maximum number of receptors that will open after the release of neurotransmitter. The correlation between both variables (*nAMPA* and *maxOPEN*) is very high (*r* = 0.98), that is, knowing *nAMPA* makes it possible to predict *maxOPEN* with a high degree of accuracy. It is important to note that the value of *nAMPA* is not directly entered in our simulated synapses, but depends on the combination of the size of the synaptic junction and the density of receptors. Indeed, we have found that the size of the synaptic junction is more influential on the postsynaptic response than the packing density of receptors. Therefore, if only the synapse size is known, *maxOPEN* can still be estimated, although with lower accuracy. By contrast, knowing [*AMPAr*] alone has no predictive value. Synaptic size is thus a relevant parameter for understanding the postsynaptic response. In practice, its importance is further enhanced by the fact that the size of large populations of synapses can be accurately measured by state of the art electron microscopy methods [5–7], while the actual number of receptors per synapse is technically very difficult to measure. Other variables such as the total apposition of extrasynaptic membranes or the concentration of glutamate transporters have little or no influence on the number of open AMPA receptors. These results are in line with previous studies showing that diffusion barriers or perisynaptic glutamate transporters have little influence on the activation of intrasynaptic AMPA receptors [34, 44].

Although we have used the actual sizes and shapes of synaptic junctions in our simulations, the concentration of postsynaptic receptors in neocortical synapses remains unknown, so it is advisable to build a population of synaptic configurations with different values for this parameter [45]. We have used a range of receptor density values comprising several extracortical sources [25], and these values have been homogeneously distributed among our simulated synapses. The actual range of receptor densities in the somatosensory cortex is, however, probably narrower, since it has been reported that AMPA receptors are expressed at similar concentrations regardless of the size of the synapse [12], and it is highly improbable that values are distributed uniformly throughout the entire range as occurs in our simulations. If the range of receptor concentrations in the neocortex were indeed narrower than the one we have used, and if its values were not distributed uniformly, then synaptic size as a regulatory mechanism of postsynaptic response would be even more important than suggested by our experiments. If, for example, in an extreme scenario, the density of receptors were constant, synaptic size would be the only parameter regulating the postsynaptic response.

We have to be cautious, however, since there is also the possibility that different populations of synapses have different densities of receptors, and/or different ranges and distributions. This is the case, for example, in the dorsal lateral geniculate nucleus where retinogeniculate and corticogeniculate synapses have different densities of AMPA receptors and different correlations between the number of receptors and the size of the synapse [16]. Another example is the hippocampus, where the density of receptors in synapses established between Shaffer collaterals and CA1 dendritic spines is much more variable than in synapses between mossy fibers and CA3 spines [11] (see also [13]). It has also been reported that in the hippocampus the density of AMPA receptors increases with the size of different populations of synapses, but with different slopes [46]. Since the actual densities and distributions of AMPA receptors in neocortical synapses are unknown, the use of a wide range of values for these parameters in our simulations is still justified, and reveals the need for experimental work on this issue.

Our results also show that the variability in the maximum number of open receptors, measured by the *cv*, is higher with smaller *nAMPA*. This phenomenon has been previously reported [28], although our data suggest a sharper decrease of variability with increasing receptor numbers. Again, the size of the synapse has a stronger influence on this effect than the density of receptors. This would imply that large synapses with a high number of postsynaptic receptors would produce stronger and more homogeneous responses, while small synapses with less receptors would produce weaker and much more variable responses. In addition to this qualitative observation, we have also quantified the *cv* of *maxOPEN* so it can be predicted from *nAMPA* with a high degree of accuracy. As happened with *maxOPEN*, if only synapse size is known, the *cv* can be estimated with lower accuracy, and knowing [*AMPAr*] alone has no predictive value. We want to stress the fact that the variability that we have observed is only due to the stochastic nature of the interaction between neurotransmitter and receptor molecules, since the number of released glutamate molecules was kept constant in our simulations.

While it is clear that increasing both the size of the synapse and the density of receptors will progressively attain a higher number of open receptors and thus stronger postsynaptic responses, the stochastic nature of neurotransmitter-receptor interactions makes it impossible to predict the exact number of receptors that will open after neurotransmitter release. It is, however, possible to calculate the probability that a given synapse reaches a certain number of open receptors (see Fig 6). This is relevant if we assume that the number of postsynaptic receptors is related to the amplitude of the excitatory postsynaptic potential (EPSP) [47] and hence to the *strength* or *weight* of the synapse. We may thus speculate that the stochastic diffusion of transmitter molecules through the synaptic cleft and/or binding to receptors may contribute to fluctuations of synaptic currents in real synapses. We must bear in mind, however, that the amplitude of the EPSP also depends on the local geometry of dendrites at synaptic input sites [48, 49], as well as on the morphology of dendritic spines and particularly on the geometry of the spine necks [50, 51]. Therefore, it is clearly necessary to further investigate the actual relationship between the number of open receptors and the EPSP in a population of synapses such as the one we have studied, which comprised a great variety of synapses on dendritic shafts and dendritic spines.

The large range of maximum number of open AMPA receptors in a realistic population of synapses with varying geometry in the same neuron also deserves attention given its possible impact on synaptic integration. One may argue that stochastic fluctuations and morphological synapse details may be averaged out during massive synaptic bombardment. However, synaptic input to cortical and hippocampal principal neurons is on a cell-assembly basis, meaning that a few synapses on single neurons are expected to co-activate frequently. The strong non-linear behavior of these dendrites [52, 53] makes them highly sensitive to the precise timing of individual inputs such that dendritic branches may fire a large number of local spikes [54], a few of which may generate a somatic spike [55]. It has been hypothesized that dendritic branches are independent computational subunits [56–58]. One may speculate that the joint modulation of groups of synapses through activity-dependent structural changes of neighboring synapses along discrete dendritic segments may constitute a functional switch for short-term plastic phenomena and the operation of computational dendritic subunits.

The present results may be of physiological relevance to the processing of information at the single cell and network levels, as well as to the possible functional role of synapses. For instance, in the cerebral cortex, the vast majority of excitatory synapses in the neuropil are formed on dendritic spines. In a recent three-dimensional electron microscopy study, we have found that over 84% of excitatory synapses are located on dendritic spines [59]. Therefore, the morphometric parameters used in the present study mostly correspond to axospinous synapses. Since synaptic size and dendritic spine size are strongly correlated [60], it has been proposed that small dendritic spines are preferential sites for long-term potentiation induction, whereas large spines might represent physical traces of long-term memory [61, 62]. We have not found a clear-cut boundary between small and large synapses either morphologically or with respect to the AMPA receptor activation response. The maximum number of open AMPA receptors increases almost monotonically with synaptic size. However, the smaller synapses in our simulated population show a sharp decrease in the coefficient of variability of response with small increments in the number of receptors, or in synaptic size. Therefore, if the function as *learning* or *memory* synapse depends only on the total number of receptors or on synaptic size, there would be a continuum distribution with no clear transition between both types. On the other hand, if the role of synapses as *learning* synapses is related to high stochastical variability, and *memory* synapses are related to low variability, the transition between learning and memory synapses would be fast. The increase of postsynaptic AMPA receptors that takes place during long-term potentiation (reviewed in [63]) could be the basis of this transition, and would help to explain the rapid establishment of changes in synaptic efficiency obtained with experimental protocols leading to plastic phenomena or dysfunction.

## Supporting Information

S1 Dataset Simulation Data. This package contains the experimental data used as basis for the analysis presented in the paper.
(ZIP)
